# New Hospitals v. The Extension of Existing Hospitals in London

**Published:** 1894-02-03

**Authors:** 


### THE INSTITUTIONAL WORKSHOP.

### HOSPITAL ADMINISTRATION.

### NEW HOSPITALS v. THE EXTENSION OF EXISTING HOSPITALS IN LONDON.

Some discussion has arisen of late on the alternative policies of (1) providing additional beds at the existing metropolitan hospitals, and (2) establishing new hospitals in outlying districts where such accommodation is much needed, as at Camberwell, which the Lords' Committee specially indicated in their report.

Critics are fond of quoting the ancient gibe against existing metropolitan hospitals that they fail to properly fulfil the functions with which they are charged owing to their being placed upon sites within a radius of two miles from Charing Cross. This fact led to a new departure in the case of the Great Northern Central Hospital, which was moved from the Caledonian Road to Islington at the instigation of the inhabitants of that district, who have loyally and liberally supported the Committee which had the courage to make the experiment. At first only half the Great Northern Central Hospital was erected, but the whole plan has now been carried out, and it is expected that the entire hospital will be open for the reception of patients by Midsummer next. The completion of this new hospital will enable the vexed question of the relative merits of rectangular and circular wards to be set at rest once and for all. It was very difficult to procure a site at all in Islington, and that chosen was so situated as to lend itself to the erection of one rectangular and one circular block of wards. Each of these blocks contains the same superficial and cubic area, the whole of the arrangements are identical, and it will be possible to treat patients under precisely similar conditions in the rectangular and circular wards, which are well worthy of inspection and study.

Reverting to the question in hand, the need for further hospital beds is made apparent by the fact that every year the population of London is increased by something like 60,000 souls. Notwithstanding this enormous annual increase in the number of those whom the managers of the metropolitan voluntary hospitals have to provide for, it appears that during the twelve months ending December 31st, 1892, out of 8,180 available beds. only 6,165 were daily occupied throughout the year, We have thus a margin of 25 per cent. of the total accommodation still available to meet the needs of the sick who seek in-patient treatment at the hospitals. These figures do not convey the whole truth, as it is necessary for the managers to keep a certain margin of vacant beds, say, 10 per cent., always available to meet special emergencies which occasionally cause a large number of accident or urgent cases to be brought in together.

There is still one other circumstance which has an important bearing upon this question of the extension of old buildings versus new hospitals. A reference to the fourth volume of "Hospitals and Asylums of the World," where plans of the sites of all the chief metropolitan hospitals are given, shows that unless further land be acquired it will not be possible to provide additional beds in new buildings at Guy's, St. Bartholomew's, St. Thomas's, St. George's, King's College, Westminster, the Royal Free, Middlesex, and Charing Cross Hospitals. We believe that the authorities of St. Bartholomew's Hospital have secured a portion of the Bluecoats' School property at enormous cost for the erection of a new nurses' home, which is most urgently needed; that at the Middlesex Hospital, with the exception of a few small houses, all the available site has been acquired and built upon; that in the case of Charing Cross Hospital the expense of acquiring an additional site so as to occupy the whole of the triangular piece of land between King William Street, Chandos Street, and Agar Street, a step most desirable on hygienic grounds, would be so great as to be prohibitory, unless a millionaire took the matter in hand; and that in the case of the other hospitals mentioned, with the exception, perhaps, of St. Thomas's, the adjacent land does not readily lend itself to the purposes of hospital extension. There remain the London Hospital, where the authorities, we understand, hold that the site available is large enough to justify the erection of a memorial wing to Sir Andrew Clark, a contention which, to say the least, is open to serious question; St. Mary's Hospital, where the governors have recently acquired a site equal in extent to about one-third of the area upon which the present hospital stands, upon which they are preparing to erect buildings to contain about 100 additional beds, and University College Hospital, the committee of which have acquired the whole of the properties at the back of the present hospital, which places them in possession of a splendid site, entirely surrounded by streets, upon which it is most desirable that there should be erected, without loss of time, a magnificent modern hospital to contain 400 or 500 beds.

The present position is, therefore, as follows: Assuming that University College Hospital is rebuilt, that additional wings are erected in connection with the London and St. Mary's Hospitals, and that the proposed extension of the cancer wards at the Middlesex Hospital proves feasible, including the Great Northern Central Hospital, some 500 beds would be added to the present accommodation provided by the metropolitan hospitals, which number might be supplemented, as an outside estimate, by 100 more beds, for which accommodation might possibly be found by reconstructions of various kinds elsewhere. From 1,500 to 2,000 other beds might be made available for London cases if admission was to be refused to all patients sent up from the country to the metropolitan hospitals for treatment. This step could never be taken in our judgment, and the idea must be regarded as impracticable.

Having thus examined the facts, let us consider what courses are open to those who desire to provide additional hospital beds for the accommodation of the sick and non-pauper poor of the metropolis. Steps may be taken (1) to erect entirely new hospitals in the centre of crowded districts like Camberwell, on the model of the Great Northern Central Hospital, which is undoubtedly proving a great success. Against this proposal we have to set the fact that the experience of the existing hospitals shows that a patient seeks admission to a hospital ward from other reasons than the circumstance that it is nearest to his place of residence. In other words, very many in-patients do travel right across London to seek admission to a particular hospital which, in point of locality, may be and often is farthest away from his own house. Further, so long as London has great medical schools, so long must care be taken to secure that the best clinical material shall be made available at every clinical hospital where students are trained. It follows that whereas new and independent hospitals might not prevent patients residing in the neighbourhood in which they may be situated from seeking admission into the older hospitals, they might, if sufficiently numerous, ultimately take away from the clinical hospitals so much valuable material as to render it difficult, if not impossible, to retain some or all of the medical schools in efficiency.

(*To be continued.*)

